# Classification challenges in the field of eating disorders: can severe and enduring anorexia nervosa be better defined?

**DOI:** 10.1186/s40337-018-0229-8

**Published:** 2018-12-10

**Authors:** Phillipa Hay, Stephen Touyz

**Affiliations:** 1 Translational Health Research Institute, School of Medicine, Western Sydney University, Sydney, Australia; 2 School of Psychology, The University of Sydney, Sydney, Australia

The field of eating disorders has notable problems of definition. For this year’s Editorial we were stimulated to focus on this because it is both the year of pre-release of the 11th Edition of the International Classification of Diseases (ICD-11) [1] and PH was invited to present a paper on recovery in eating disorders. In doing so it became clear that there is little consensus for definitions for recovery [2] or more broadly other important clinical phases of anorexia nervosa. In particular, definitions of severe and/or enduring illness are elusive and the concept of ‘severe and enduring’ is frequently misunderstood in terms of its conceptualisation [3, 4]. Such lack of definitional clarity is a problem for researchers, but even more so for clinicians who become confused, and most importantly for those living with the eating disorder.

Despite lack of definitional clarity, people with unremitting anorexia nervosa have very poor outcomes [5, 6] and there is little evidence based treatment for their care [7, 8]. Only in the twenty-first century was an attempt made to incorporate recommendations for this group into clinical practice guidelines [8]. However, these guidelines referred to implicit clinical descriptors of longstanding illness with high morbidity, poor function and high health care use. In contrast diagnostic schemes such as the ICD-11 and Diagnostic and Statistical Manual of Mental Disorders Fifth edition (DSM-5) [9]) have explicit threshold criteria for persons to achieve a categorical diagnosis. The guidelines [8] did not apply or suggest specific criteria for severe and enduring anorexia nervosa - as there are none agreed – or discuss other severe and enduring eating disorders.

Clear definitions are very important. They are the reason the DSM-III in 1980 was universally popular to the point that copies had to be chained to desks to prevent their theft by students in the hospitals where we (PH and ST) then worked. The understanding of aetiology, prognosis and the relationships between disorders rests on precise diagnosis, and valid and reliable diagnostic systems are necessary for meaningful communication between clinicians and researchers [10]. Let’s examine ‘severe and enduring’ anorexia nervosa as it is conceptualised and then as it may be defined. We purport there are three components of severe and enduring anorexia nervosa: persistent unremitting symptoms, of long duration, and treatment resistance. First, the DSM-5 has definitions of severe for anorexia nervosa based on levels of body mass index (BMI; kg/m^2^) which are cross-sectional [9]. Dakanalis et al. found these definitions of severity predict or converge with other aspects of anorexia nervosa illness severity and functional impact [11]. However, in the case of unrelenting illness these should be tempered as a person with a decade or more years of a BMI above the DSM-5 severe range will have considerable morbidity from chronic starvation and longstanding illness – the latter term applied in the Touyz et al. randomised controlled trial of treatments for this group [7]. Second, considering duration, a systematic review reported that most who have written about this state in anorexia nervosa are referring to more than a few years of experiencing diagnostic level eating disorder symptoms with several previously failed treatment attempts [4]. The number of years of illness in the published literature ranges from 3 to 10, with a mode of 7 in this review [4]. In sum, the agreed lower limit of years appears to be three. The third key feature of treatment resistance is the most problematic to define. There are a diversity of approaches where individuals and/or their families have not responded to or do not wish to engage in first line therapies and also major differences across and often even within regions in how, when and what treatments patients are able to access.

As a trainee psychiatrist I (PH) recall a common question in our examinations was “The specialist approach to assessment of a person with treatment resistant disorder X”. The answer always began with first, review the diagnostic formulation. Only after establishing that that the formulation is accurate, and the person has been offered and received all appropriate first line evidence based therapy is the term “treatment resistant” to be applied. Then the specialist will discuss second and third line therapies, including those often with higher risk, e.g., clozapine for schizophrenia or irreversible monoamine oxidase inhibitors for major depression. Finally, the “do no harm” or “comfort always” time honoured approach of clinical medicine – supportive care – may be offered.

However, we are reluctant to use the term “treatment resistant” in our field. It is perceived understandably as pejorative and blaming of the individual and those who care for them. It may seem to imply that as we have evidence based therapies, it’s the person’s fault if they “drop out”, do not engage or otherwise are unresponsive. On the other hand, they may have not engaged because of poor health literacy on the part of the health care practitioner [12] who has e.g., told them when they sought help that they are not thin enough to have an eating disorder [13]. With the best of efforts treatment may yet fail if it is not person or family centred but rather ‘Somebody Else’s Roadmap’ [14]. Treatment resistance is also poorly understood with no consensus on e.g., how many attempts at re-feeding or other treatments are enough before they cease. Yet, it is a common predicament for clinicians and people with the lived experience of anorexia nervosa.

Finally, it must be acknowledged that categorical systems are problematic in mental health where clinical features are often dimensional and boundaries are by necessity somewhat arbitrarily imposed. In consequence, diagnostic schemes such as the ICD-11 [1] allow more clinician judgment and flexibility than the DSM-5 [9] and may thus reduce the likelihood of harm from over rigid application of criteria in individual cases. As Wonderlich et al. [15] pointed out there is no strong empirical support for a clear demarcation separating people with longstanding anorexia nervosa from others. Thus, whilst we propose testable criteria (see Table 1) for ‘severe and enduring anorexia nervosa’ we would advise their judicious use by practitioners mindful of their limitations.

**Table 1:** Proposed criteria for “severe and enduring anorexia nervosa”

(1) a persistent state of dietary restriction, underweight, and overvaluation of weight/shape with functional impairment	
(2) duration of > 3 years of anorexia nervosa; and	
(3) exposure to at least two evidence based treatments appropriately delivered together with a diagnostic assessment and formulation that incorporates an assessment of the person’s eating disorder health literacy and stage of change	

The criteria we propose are based on (1) clinically significant functional impact i.e. impoverished and poor quality of life, with unrelenting symptoms, i.e. sustained dietary restriction leading to a persistent underweight state with weight/shape overvaluation and other eating disorder cognitions (2) duration of several years (minimum three) of anorexia nervosa, and (3) exposure to at least two evidence based treatments delivered by an appropriate clinician or treatment facility together with a diagnostic assessment and formulation that incorporates an assessment of the person’s eating disorder health literacy with an assessment of the person’s stage of change.

In conclusion, the Journal of Eating Disorders encourages submissions on any aspect of improving and testing current classifications and definitions in any domain relevant to the field, and including the neurosciences, in the spirit of the Research Domain Criteria approach [16]. We look forward to publishing research that will better define severe and enduring anorexia nervosa, recovery and other clinical states broadly in the field.
